# Comparative transcriptomic responses of tomato to *Xanthomonas* strains isolated from different hosts

**DOI:** 10.1371/journal.pone.0361370

**Published:** 2026-11-02

**Authors:** Lorena Sánchez-Giménez, Eugenio Llorens, Atefeh Farvardin, Jaime Cubero, Loredana Scalschi, Begonya Vicedo, Gemma Camañes

**Affiliations:** 1 Departamento de Biología, Bioquímica y Ciencias Naturales, Grupo de Bioquímica y Biotecnología, Universitat Jaume I, Castellón de la Plana, Spain; 2 Instituto Nacional de Investigación y Tecnología Agraria y Alimentaria (INIA), Consejo Superior de Investigaciones Científicas (CSIC), Madrid, Spain; NSTRI: Nuclear Science and Technology Research Institute, IRAN, ISLAMIC REPUBLIC OF

## Abstract

Bacterial spot disease, caused by several *Xanthomonas* species, poses a significant threat to tomato and pepper production worldwide. Among these pathogens, *Xanthomonas vesicatoria* and *Xanthomonas euvesicatoria* pv. euvesicatoria have emerged as particularly important pathogens due to their widespread distribution and economic impact. While both pathogens cause similar symptoms, their genetic and pathogenic differences influence infection dynamics. In this study, we conducted a comparative transcriptomic analysis of tomato plants infected with one strain of *X. vesicatoria* (isolated from tomato) and one of *X. euvesicatoria* pv. euvesicatoria (isolated from pepper) to elucidate differential gene expression patterns associated with tomato defense responses. RNA sequencing at 48 hours post-infection revealed a higher number of differentially expressed genes (DEGs) in response to *X. vesicatoria*, with significant enrichment in pathways related to cell wall modification, secondary metabolism, and phytohormone signaling. On the other hand, infection with *X. euvesicatoria* pv. euvesicatoria induced a more limited transcriptomic response, with upregulation of a lesser number of genes involved in stress response. Key defense-related genes, including pathogenesis-related proteins (PR1, PR2, PR5), chitinases, and peroxidases, were activated in both infections but showed higher expression levels in response to *X. vesicatoria*. These findings suggest that *X. vesicatoria* elicits a stronger immune response in tomato plants than *X. euvesicatoria* pv. *euvesicatoria* under the conditions tested. This differential response likely reflects distinct interaction strategies employed by these strains during host colonization. Overall, our study provides new insights into the molecular mechanisms underlying tomato-*Xanthomonas* interactions and identifies potential molecular targets for developing bacterial spot-resistant cultivars.

## Introduction

The genus *Xanthomonas* includes many bacterial pathogens that can affect the productivity of economically important crops. Among them, *Xanthomonas vesicatoria* (Xv) and *Xanthomonas euvesicatoria* pv. euvesicatoria (Xe) are prominent pathogens responsible for bacterial spot disease in tomato (*Solanum lycopersicum*) and pepper (*Capsicum* spp.), causing necrotic lesions on the leaves, stems and fruits, and resulting in significant yield and quality losses under favorable environmental conditions [1,2]. Management of bacterial spot relies on integrated strategies, including the use of pathogen-free seeds, cultural practices and copper-based bactericides [2]. However, disease control remains challenging due to the emergence of resistant bacteria and the limited effectiveness of available control measures. Disease symptoms can be similar for both bacterial species, making their differentiation challenging without precise identification of the strain, mainly through molecular approaches. Understanding the main differences between Xv and Xe may be critical for improving disease management and the success of developing new resistant cultivars [3].

One of the most notable differences between *Xanthomonas* spp. is their variability in host association and environmental adaptation [2,4]. These bacteria exhibit marked host specificity, with some *Xanthomonas* spp. and pathovars highly adapted to infect a specific plant host. Nevertheless, this specificity is not absolute, and a remarkable dichotomy can be observed. While some members of this genus display a narrow host range, infecting only certain plant species, others are capable of causing disease in a diverse array of taxonomically different hosts [5,6]. On the other side of this dichotomy, some plant species may be host to infection by closely related as well as phylogenetically distant *Xanthomonas* spp., highlighting the complexity and unpredictability of host-pathogen relationships within this genus.

The classification and taxonomy of *Xanthomonas* spp. infecting tomato and pepper have evolved substantially, progressing from an initial single-species grouping to the current recognition of multiple distinct species, differentiated on the basis of genotypic, phenotypic, and pathogenicity-related traits [2,7]. Specifically, Xv and Xe represent distinct taxonomic lineages that differ in their biochemical and genetic characteristics. Moreover, the Xv clade shows greater genetic heterogeneity compared to the more clonal population of Xe, which presents a narrower genetic pool but with a broader geographical distribution [8,9]. These genetic and phenotypic differences may contribute to distinct interaction strategies with both the host plant and the surrounding environment [5,10].

Despite its relatively clonal population structure, Xe exhibits high genomic plasticity driven by high plasmid diversity, horizontal gene transfer and variation in effector repertoires. This genetic flexibility contributes to the variability in virulence-associated traits, including those involved in host interaction and responses to environmental conditions. In contrast, Xv seems to be more commonly associated with tomato, where it can cause significant crop losses in susceptible varieties [5,11]. Host specificity is likely driven by the ability of pathogens to produce and use virulence factors that help in the colonization of a specific host [9]. Variation in genes encoding virulence factors, such as type III secretion system (T3SS)-associated effectors and adhesion molecules, can also indicate differences in the host specificity and the pathogenic potential between Xv and Xe [6,12–15]. Beyond genetic differences, Xv and Xe can also be slightly differentiated based on their biochemical traits [16]. For instance, Xv displays positive amylolytic activity and oxidative-fermentative metabolism, whereas Xe is negative for amylase but shows lipase activity [17,18]. These differences in the metabolic capabilities can influence their survival and virulence in distinct host plants, as some enzymes are required for an effective colonization of the host tissue [19].

On the other hand, plants have developed a variety of sophisticated and wide array of defense strategies to protect themselves against the constant threat of pathogens [20]. Plant’s immunity is based in a two-layered immune system which also involves complex transcriptional reprogramming, in which a set of specific genes are up-regulated or down-regulated once the pathogen is perceived [21]. The first layer involves pathogen recognition receptors (PRRs) that detect conserved microbial patterns, activating pattern-triggered immunity (PTI). The second layer, effector-triggered immunity (ETI), is activated upon recognition of specific pathogen-derived effectors, such as those delivered by the T3SS [20]. These mechanisms form an intricate network that enables plants to effectively recognize and respond to infections, although pathogens often evolve strategies to evade or suppress these defenses. For instance, plant basal immunity has been reported to be suppressed by the exopolysaccharide xanthan, whose production is primarily controlled by the gum operon, which is highly conserved among *Xanthomonas* spp. [22]. Currently, some studies are focused on identifying resistance genes in tomato plants and characterizing the signaling pathways that can be involved in the plant immune response, particularly those based on PRRs and T3SS-induced responses. However, these studies on plant responses are still limited and the updated taxonomic classification has rarely been integrated. This becomes particularly relevant considering that an accurate understanding of plant-pathogen interactions, within the framework of this new classification, is crucial for effective biocontrol. Due to their host-specific interactions, breeding programs need to consider the distinct pathogenicity profiles for each species [23,24]. For instance, resistance genes that confer protection against Xe on pepper may not be effective against Xe in tomato, and vice versa [4,25]. Furthermore, the development of resistant cultivars is further complicated by the ability of these pathogens to evolve new virulence strategies through horizontal gene transfer and recombination, potentially leading to the emergence of novel pathogenic strains with increased aggressiveness [6,16,26].

Based on the studies conducted so far on Xv and Xe, this work proposes a comparative study of the response of a susceptible tomato variety to the two species of *Xanthomonas*. We focused on the plant’s early response to strain CFBP 1941 of Xv originally isolated from tomato, and strain CFBP 8114 of Xe originally isolated from pepper. The objective was to elucidate how tomato plant’s defense mechanisms varied when challenged by Xv compared to Xe, by highlighting key aspects of the plant’s early immune response, including defense-related signaling pathways and the activation of pathogenesis-related genes.

## Materials and methods

### Microbial strains, growth conditions and plant material

*Xanthomonas vesicatoria* CFBP 1941 isolated from tomato and *Xanthomonas euvesicatoria* CFBP 8114 isolated from pepper were used in this study (Collection Francaise de Bacteries Phytopathogenes). Glycerinates maintained at −80 °C were grown in Nutrient Agar (NA) medium (Scharlab S.L., Spain) and incubated at 28 °C for 24–72 h, re-streaking the bacteria before the experiment.

Tomato plants *Solanum lycopersicum* cv. Moneymaker (MM) were grown in fine vermiculite and irrigated with Hoagland solution at pH 6 [27] for 5-weeks before inoculation. Plants were maintained in growth chamber under controlled conditions with day and night temperatures of 24 °C and 18 °C, respectively, with 16 h of light (200 μmol/m^2^·s) and 8 h of darkness, and 65% relative humidity (RH).

### Microbial bioassays

For inoculation, Xv and Xe strains were grown in NA at 28 °C for 48 h. Initially, the inoculum concentration for both bacteria was adjusted to 5·10^6^ CFU/mL. However, preliminary inoculations with Xe showed very mild and delayed symptoms in comparison to Xv. Therefore, to obtain comparable levels of disease development, the final bacterial suspensions were adjusted to 5· 10^6^ CFU/mL and 1· 10^5^ CFU/mL for Xe and Xv, respectively, in sterile MgSO4 (10 mM) containing 0.01% of the surfactant Silwet L-77 (Osi Specialties, USA). Tomato plants were divided into three groups of six plants each: one group was inoculated with Xv, another with Xe, and the third group consisted of non-inoculated plants (control). Pathogen inoculation was performed by dipping 3rd and 4th leaf into the bacterial suspension. Plants, including controls, were maintained at 24 °C with 16 h of light and 8 h of darkness and 100% RH. Inoculated leaves from each plant were collected at 48 h post-infection for RNAseq analysis.

Disease rating and colony counting were performed at 5 days post-infection. Colony counting was performed from the inoculated leaves of infected plants homogenized with MgSO_4_ 10 mM 1% p/v. Serial dilutions were deposited by drops and colony counted 48 h later in NA with selection antibiotics (cephalexin 65 mg/L, 5-fluorouracil 12 mg/L) and the antifungal cycloheximide 50 mg/L. Statistical significance was determined in RStudio using Student’s t-test (p < 0.05). Data distributions were visualized using box plots.

### RNA sequencing (RNA-seq) and bioinformatic analysis

Total RNA was extracted from the inoculated and control leaf samples collected 48 h post-inoculation, using the RNeasy Kit for RNA Purification (Qiagen, Germany) according to the manufacturer’s instructions. Total RNA was extracted from non-inoculated plants and from plants inoculated with either Xv or Xe, with three biological replicates per condition. The concentration of total RNA was evaluated before RNA-seq using a NanoDrop 2000 spectrophotometer (Thermo Fisher Scientific, USA). Moreover, to ensure the suitability of the RNA for RNA-seq, only samples exhibiting an RNA Integrity Number (RIN) greater than 7.0, calculated with an Agilent QC System, were included in subsequent analyses. Extracted RNAs were sent to Macrogen, Inc. (Seoul, Republic of Korea) and sequenced on an Illumina NovaSeq 6000 platform with 151 bp paired-end reads. RNA libraries were prepared using the TruSeq Stranded Total RNA Library Prep Plant Kit and the sequencing depth was targeted at 40 million reads per sample for comprehensive coverage as recommended in transcriptomic analysis of tomato. Raw reads were quality-filtered using FastQC v0.11.7 (http://www.bioinformatics.babraham.ac.uk/projects/fastqc/) and Trimmomatic for trimming and preprocessing high-throughput sequencing data [28].

Raw reads were aligned to the tomato reference genome SL3.1 using HISAT2 version 2.1.0 (https://ccb.jhu.edu/software/hisat2/index.shtm) and StringTie version 2.1.3b was used to estimate the expression levels of the transcripts based on the read count value of known genes. Expression profile was calculated for each sample and transcript/gene as read count, FPKM (Fragment per Kilobase of transcript per Million mapped reads) and TPM (Transcripts per Kilobase Million). Genes with more than one zero read count across the nine samples were excluded from further analysis.

Clean data from this work has been submitted to the National Center of Biotechnology Information (NCBI) Sequence Read Archive under accession number BioProject ID PRJNA1288707.

Differentially Expressed Genes (DEGs) analysis was performed on the two comparison pairs: Xe *vs*. control and Xv *vs*. control, using DESeq2 on raw count data. Genes were considered differentially expressed based on a fold change (FC) ≥ 2 and a p.value < 0.05. DEG lists, volcano plots, and hierarchical clustering analyses were generated based on these criteria. Subsequently, DEG lists were analyzed with g:Profiler (https://biit.cs.ut.ee/gprofiler/orth) for gene set enrichment analysis of biological process (GO:BP), cellular component (GO:CC) and molecular function (GO:MF). Enriched GO terms were considered significant based on an adjusted p.value (false discovery rate, FDR) < 0.05. To compare the expression profiles between Xv- or Xe- inoculated samples and control plants, similarity between samples was assessed using Pearson’s correlation coefficient calculated from rlog-transformed expression values.

Hierarchical clustering with Euclidean distance was performed on DEGs to determine the similar expression patterns between samples and genes.

### qRT-PCR validation

For transcriptomic validation, qRT-PCR was performed with the RNA samples isolated as previously described. 1 μg of total RNA was reverse-transcribed into cDNA using PrimeScript RT Reagent Kit (Perfect Real Time; Takara, Japan). Quantitative real-time PCR was performed with Maxima SYBR Green/ROX qPCR Master Mix using the StepOneTM Real-Time PCR System (Thermo Fisher Scientific, USA).

Primers for divinyl ether synthase (*DES*), pathogenesis-related protein STH-2 (*STH2*), pathogenesis-related protein P2 (*PR-P2*), 3-ketoacyl-CoA synthase (*3KCoAS*), GDSL esterase (*GDSL*), U1 spliceosomal RNA (*U1S*) and the elongation factor *EF1a* (housekeeping gene) were designed using Primer3web and synthetized by IDT (www.idtdna.com). Differences in cycle numbers during the linear amplification phase between samples from infected and control plants were used to determine differential gene expressions. Relative quantification was performed using standard curves for each target gene. The corresponding slope, R² and amplification efficiency values are provided in S1 Table. Expression levels of each gene in individual samples were normalized to the reference gene *EF1α*. The following primers were used: *DES*-Fw (5’-ACGAGACATTGAGACTTCGC-3’) and *DES*-Rv (5’-CAGGTACAAACTCATCCGGG-3’); *STH2*-Fw (5’- ACACCAGTTGCCCCTACTAG-3’) and *STH2*-Rv (5’- TGGTCACCAAATTCTTGTCATCA-3’); *PRP2*-Fw (5’- GCTACCTGGGATGCTGACAA-3’) and *PRP2*-Rv (5’- CAAGCCCTCCATTTCTGCAT-3’); 3*KCoAS*-Fw (5’-TGTGTTACCCCTTACTGAGCA-3’) and 3*KCoAS*-Rv (5’-TAATACTGCCCTTCCTCCCG-3’); *GDSL*-Fw (5’-ACGTTTGGGGATTCAGCTTC-3’) and *GDSL*-Rv (5’-CCGGGTAAGTAGTGAAGCCA-3’); *U1S*-Fw (5’-TCAATGGGCGATCAATAAGGC-3’) and *U1S*-Rv (5’-AGCAACAAATTATGACGTAGGCT-3’); *EF1a*-Fw (5’-GACAGGCGTTCGGTAAGGA-3’) and *EF1a*-Rv (5’-GGGTATTCAGCAAAGGTCT-3’).

## Results

### *Xanthomonas* infection in tomato plants

To evaluate the changes induced in the tomato during the infection by both *Xanthomonas* spp., five-week-old tomato plants (*S. lycopersicum* cv. Moneymaker) were used due to their high susceptibility. During the optimization of the leaf infection assays, several inoculum concentrations were tested. To standardize symptom severity between the two strains, inoculum concentration of 1· 10^5^ CFU/mL for Xv and 5· 10^6^ CFU/mL for Xe were selected*.* Disease symptoms evaluation (Fig 1A and 1C) and bacterial population recovery (Fig 1B) were conducted at 5 days post-infection. The inoculum concentration of Xv was reduced from its initial value due to excessive aggressiveness, to achieve a symptom severity comparable to that caused by Xe and allow a balanced comparison of their effects on the plant fitness.

**Fig 1:**
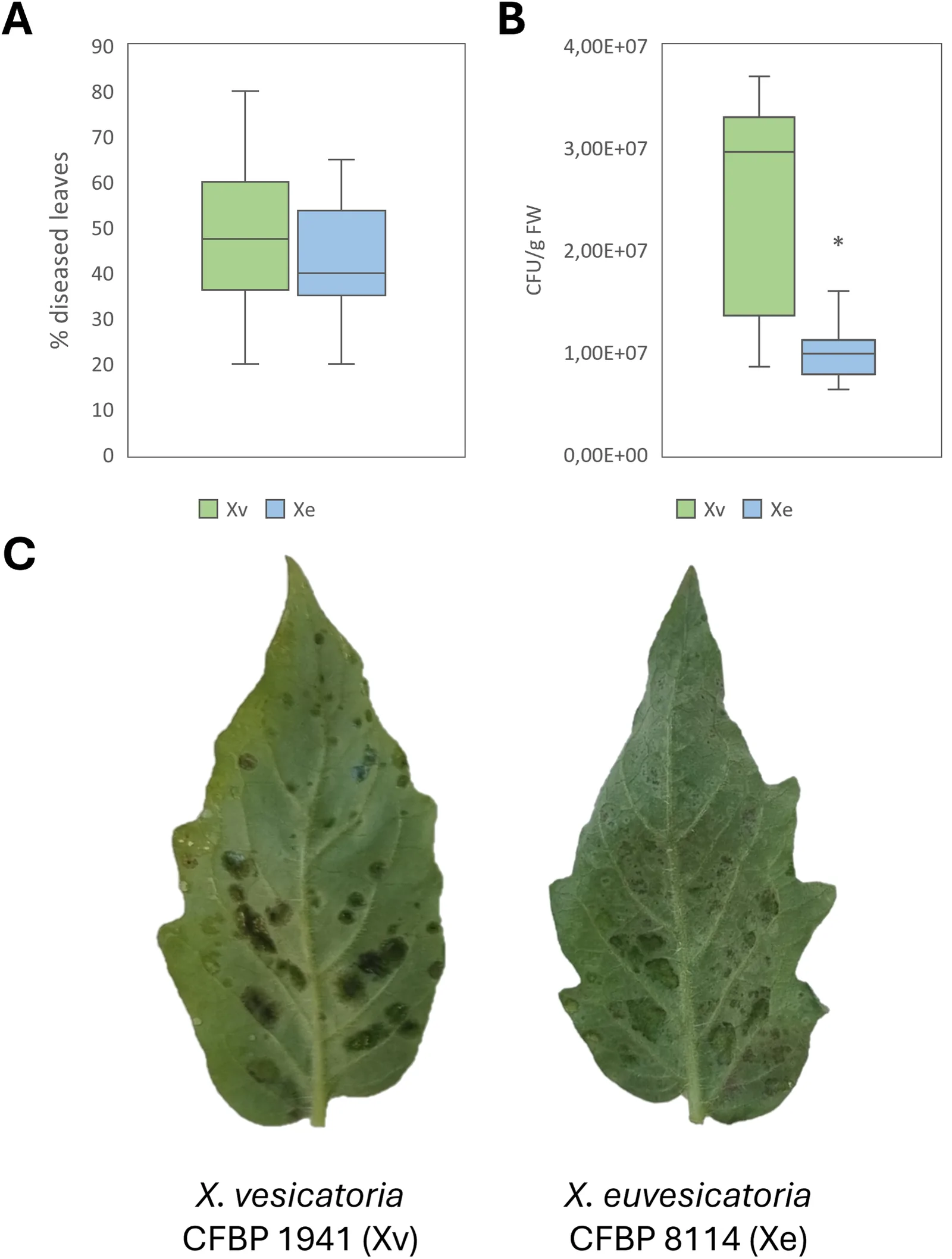
Bacterial recovery and symptoms evaluation from Xv and Xe in tomato plants. (A) Disease rate scored after 5 days of inoculation by percentage of dark-brown spots on the leaf surface and its severity. (B) Bacterial colonization by bacterial counting on NA medium with selection antibiotics (FW = fresh weight). (C) Representative image of the leaf damage on infected plants either with Xv or Xe. Graphs (A, B) display box plots with the median and interquartile range (n = 6). Asterisk represents statistically significant differences (p < 0.05; t-student test).

During the initial stages of symptom development, circular water-soaked lesions appeared, which later dried into darker spots over time. However, the severity of the leaf damage was greater in plants infected with Xv than in those infected with Xe, despite the lower inoculum. The necrotic lesions caused by Xv were deeper and more severe, extending throughout the leaflet. In contrast, Xe produced more superficial lesions, with extensive water-soaking being observed at times, as previously described for this bacterium [1]. Bacterial counts also revealed that the population of Xe on the inoculated tomato leaves was significantly lower than that from Xv*-*infected plants.

### Analysis of RNA-seq libraries and quality of the samples

To obtain a global overview of the transcriptome relevant to Xv and Xe infections in tomato plants, the cDNA libraries from six inoculated and three mock samples were sequenced. Sequencing generated from 40.2 to 59.6 million reads from each sample, with a quality score of ≥ Q20 for 97% of the raw reads. Clean reads ranging from 39.4 to 58.3 million per sample were obtained after filtering (S2 and S3 Tables) and mapped to the tomato reference genome (S4 Table). The coverage of mapped reads was in all the samples over 84%. To ensure data quality, gene samples with more than one read count equal to 0 were not included in the analysis. Therefore, from a total of 30,824 genes, 13,700 were excluded and only 17,124 genes were used for further statistical analysis.

### Identification of DEGs in infected and non-infected plants

Different expression analysis was performed on the two comparisons pairs. Results showed 933 genes which satisfied a |FC| ≥ 2 and p-value < 0.05 conditions in at least one of comparison pairs and were considered DEGs. The hierarchical clustering analysis based on each sample’s rlog-transformed values grouped samples with similar expression profiles. Results suggest that Xe-infected plants and control plants exhibit a smaller distance between them, while Xv-infected plants form a distinct cluster on a separate branch (Fig 2).

**Fig 2:**
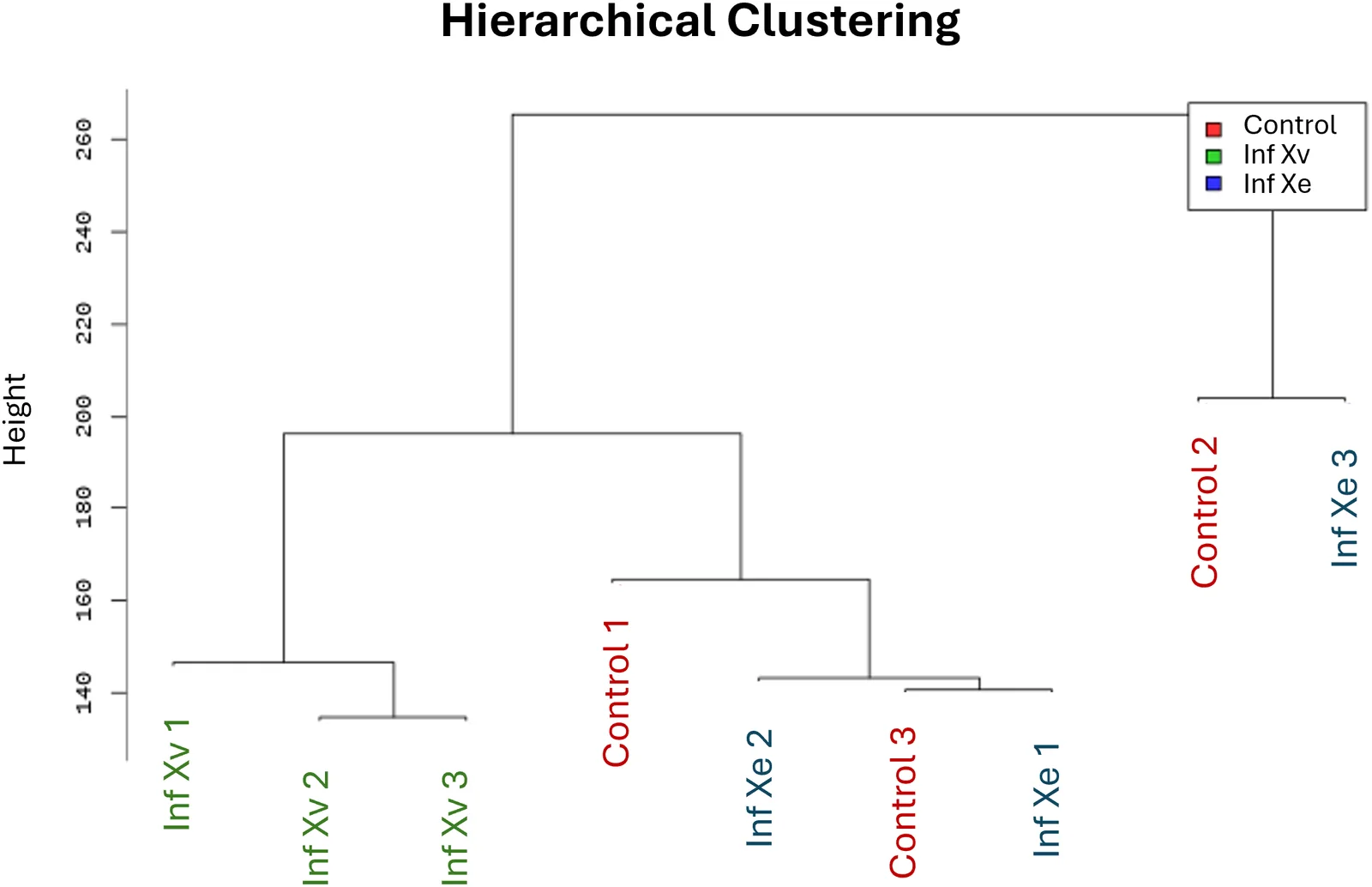
Hierarchical clustering of RNA-seq samples from control plants, Xv-infected plants and Xe-infected plants based on rlog-transformed gene expression values. Samples with similar global expression profiles clustered together. Hierarchical clustering was performed using Euclidean distance as the distance metric and complete linkage.

Subsequently, gene-set enrichment analysis was performed on DEGs. Specifically, 765 DEGs were identified in Xv infected plants compared to the control, with 540 upregulated and 225 downregulated. In contrast, the number of DEGs in Xe infected plants were lower, with only 409 DEGs, including 316 upregulated and 93 downregulated (Fig 3).

**Fig 3:**
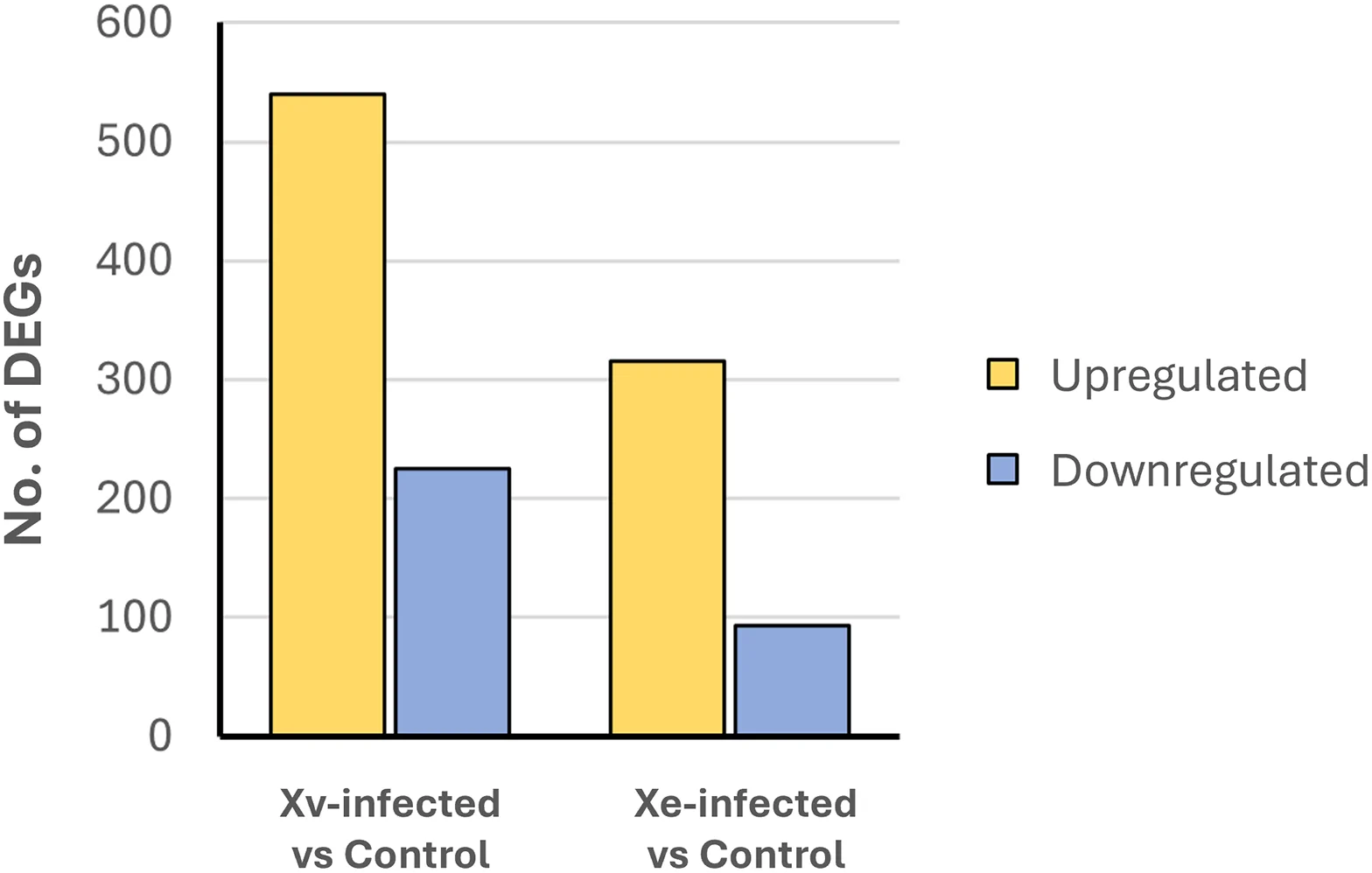
Number of DEGs identified in tomato plants infected with Xv or Xe compared with non-inoculated control plants. Graphs represent the number of genes that were found to be upregulated (yellow bars) or downregulated (blue bars) at 48h post-inoculation with each bacterium.

Volcano plots revealed differential gene expression between infected and control plants for both Xv and Xe*-*infected samples*.* Overall, inoculation with Xv resulted in a greater number of tomato plant genes showing higher |FC| values and stronger statistical significance compared to the gene expression profile observed in plants infected with Xe (Fig 4).

**Fig 4:**
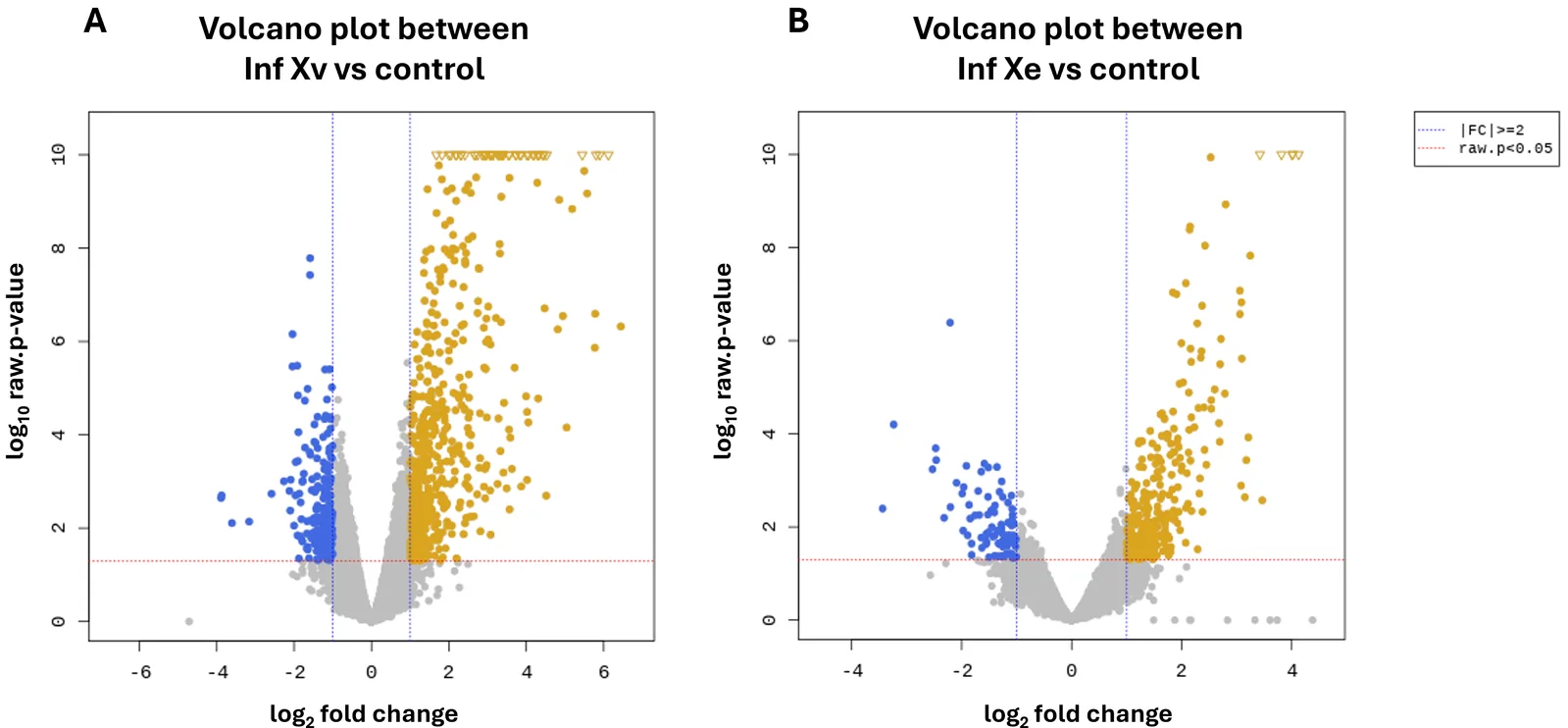
Volcano plot between Xv-infected plants (A) and between Xe-infected plants (B) compared to control plants. Yellow dots represent upregulated genes, whereas blue points represent downregulated genes (|FC| ≥ 2, p < 0.05).

Of the total genes upregulated in response to the infection with either Xv or Xe, only 33% (213) were common for both pathogens. Infection with Xv induced a stronger response with 327 genes uniquely upregulated compared to control plants, whereas 103 genes were uniquely upregulated in response to Xe infection. Similarly, the highest number of downregulated genes resulted from plants infected with Xv, accounting 197 genes (67% of the total), while 65 genes (22% of the total) were uniquely downregulated in plants inoculated with Xe (Fig 5).

**Fig 5:**
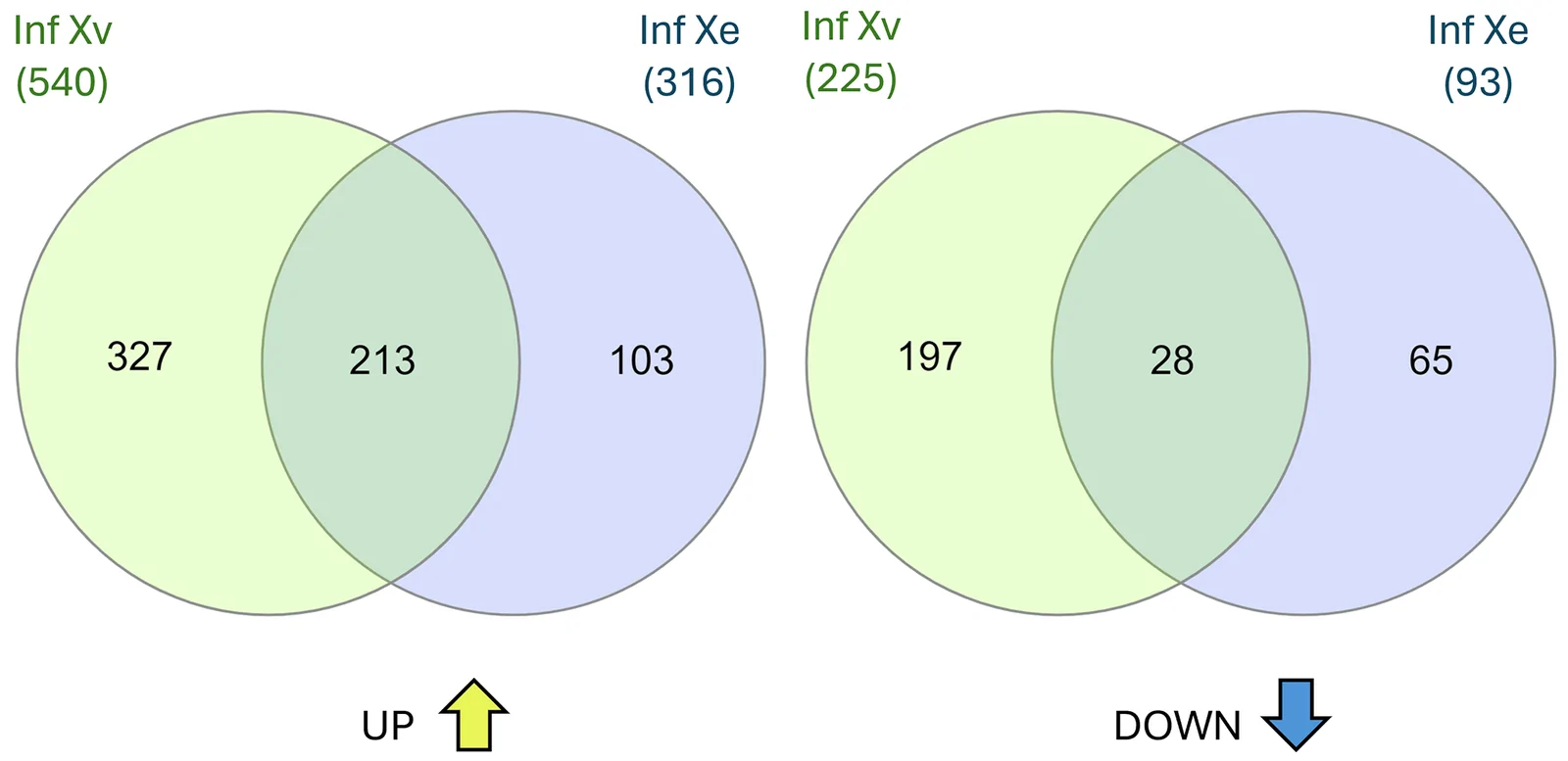
Venn diagram represents the number of DEGs in tomato plants infected either with Xv or Xe vs. control plants. Genes upregulated are represented on the left side, while genes downregulated after infection are represented on the right side. Total values for each infection are also shown.

### Validation of transcriptomic analysis by qRT-PCR

To validate the transcriptomic results, qRT-PCR analysis was performed on six genes that were identified as differentially expressed during the infection with both bacteria: *DES*, *PR-P2*, *STH2*, *3KCoAS, GDSL* and *U1S* using *EF1a* as internal reference. The qRT-PCR results were consistent with the RNA-seq data, confirming the tendency on the differential expression patterns observed in the transcriptomic analysis (Fig 6).

**Fig 6:**
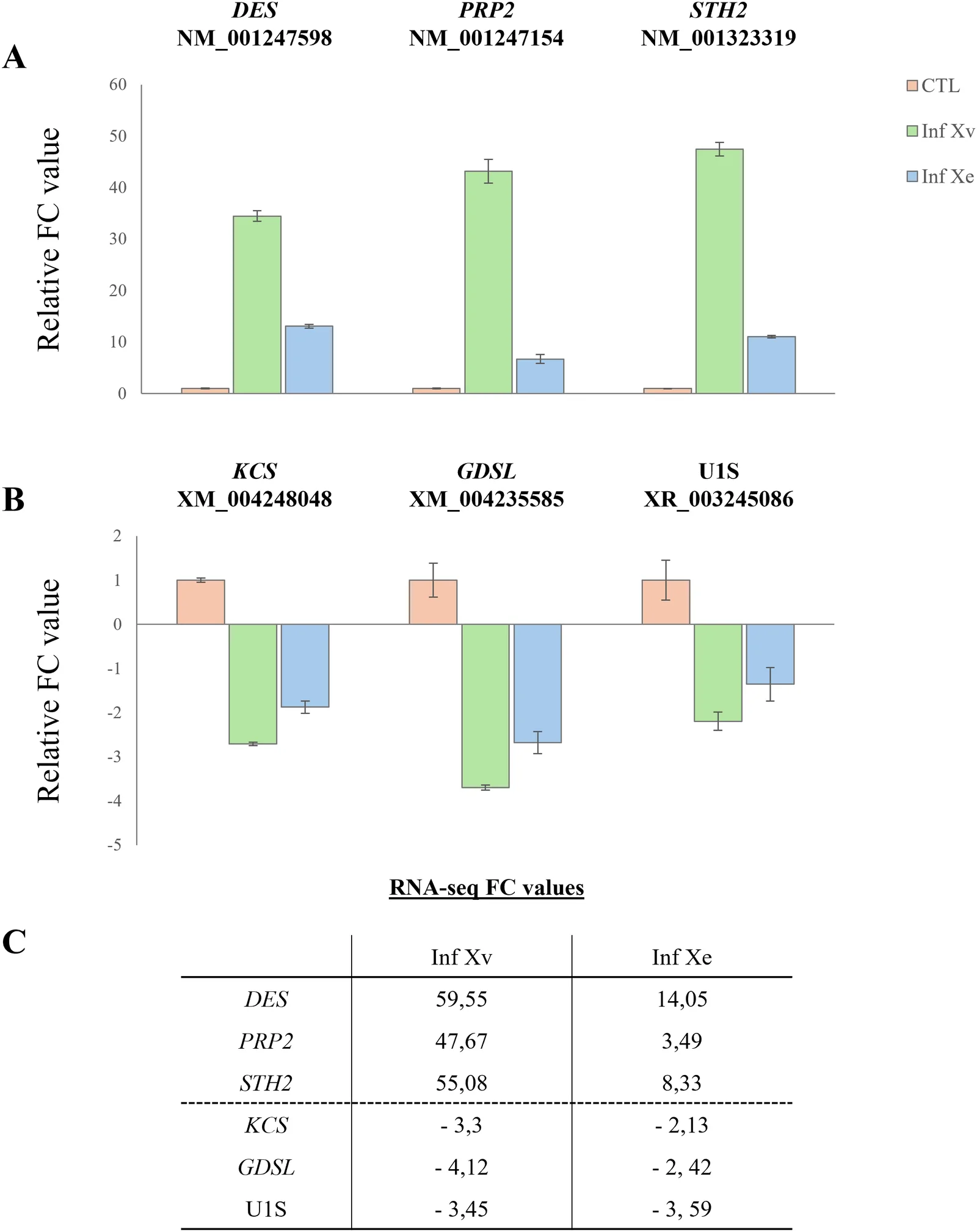
RT-qPCR validation of DEGs related to defense response in Xv and Xe infection compared to control plants. For RT-qPCR results, constitutive gene *EF1a* was used as a housekeeping gene. Fold change values over control plants of (A) up-regulated genes or (B) down-regulated genes are represented. (C) FC values in RNA-seq are displayed on the table. Results show the average of three independent experiments with similar results ± standard error (n = 3).

### Gene Ontology (GO) classification

To investigate the biological processes associated with the DEGs, a total of 175 of them from Xv-infected plants and 128 from Xe-infected plants were functionally categorized based on their similarity to known genes in reference databases and classified according to their putative involvement in various biological processes. Subsequent enrichment analysis using the g:Profiler tool and Uniprot (https://www.uniprot.org/) revealed significant over-representation of specific Gene Ontology (GO) terms, biological pathways, and interaction networks among the DEGs (Table 1).

**Table 1:** Classification of DEGs into GO terms related to Biological Process (GO:BP) during the infection response to Xv or Xe.

	GO Code	Biological process	Adj. p.value	Up-regulated	Down-regulated	Total
**DEGs in Xv-infection**	GO:0046348	Amino sugar catabolic process	6.14E-06	7	0	7
	GO:0006022	Aminoglycan catabolic process	1.04E-05	7	0	7
	GO:0042537	Benzene-containing compound metabolic process	3.34E-04	4	2	6
	GO:1901136	Carbohydrate derivative catabolic process	6.22E-06	8	1	9
	GO:0005975	Carbohydrate metabolic process	8.86E-03	15	13	28
	GO:0009056	Catabolic process	1.22E-02	25	9	34
	GO:0044036	Cell wall macromolecule metabolic process	1.16E-02	6	2	8
	GO:0071554	Cell wall organization or biogenesis	2.03E-02	8	7	15
	GO:0006032	Chitin catabolic process	6.14E-06	7	0	7
	GO:0009805	Coumarin biosynthetic process	3.19E-02	4	0	4
	GO:0009835	Fruit ripening	4.50E-02	3	0	3
	GO:1901072	Glucosamine-containing compound catabolic process	6.14E-06	7	0	7
	GO:0006591	Ornithine metabolic process	4.50E-02	3	0	3
	GO:0018958	Phenol-containing compound metabolism	1.01E-03	3	2	5
	GO:0009699	Phenylpropanoid biosynthetic process	4.98E-04	7	0	7
	GO:0030639	Polyketide biosynthetic process	2.73E-02	0	2	2
	GO:0009620	Response to fungus	1.22E-02	8	0	8
	GO:0009696	Salicylic acid metabolic process	4.98E-04	3	2	5
	GO:0019748	Secondary metabolic process	3.34E-04	7	3	10
		Total				175
**DEGs in Xe*-*infection**	GO:0046349	Amino sugar catabolic process	1.29E-06	5	1	6
	GO:0006023	Aminoglycan catabolic process	2.15E-06	5	1	6
	GO:0044419	Biological process involved in interspecies interaction between organisms	1.88E-02	8	2	10
	GO:1901137	Carbohydrate derivative catabolic process	8.86E-05	5	1	6
	GO:0044037	Cell wall macromolecule metabolic process	5.40E-03	5	1	6
	GO:0006033	Chitin catabolic process	1.29E-06	5	1	6
	GO:0009806	Coumarin biosynthetic process	2.59E-02	2	1	3
	GO:0006952	Defense response	1.88E-02	13	1	14
	GO:1901073	Glucosamine-containing compound catabolic process	1.29E-06	5	1	6
	GO:0009699	Phenylpropanoid biosynthetic process	1.19E-03	4	1	5
	GO:0009607	Response to biotic stimulus	8.06E-03	9	2	11
	GO:0009621	Response to fungus	8.13E-04	5	2	7
	GO:0050896	Response to stimulus	6.90E-03	28	9	37
	GO:0019748	Secondary metabolic process	2.28E-02	4	1	5
		Total				128

Adjusted p.value and number of upregulated and downregulated genes compared to control for each biological process. Complete data provided in S1 and S2 Files.

Plants infected with Xv exhibited an enrichment of genes involved in secondary metabolism biosynthetic processes, as well as with a reorganization in the expression of genes associated with cell wall organization or biogenesis, an essential first layer of defense against invading pathogens. More specifically, DEGs related to the metabolism of ornithine, coumarin or salicylic acid metabolism were observed after infection, all of which play roles activating the immune system against pathogen attack. Other biological processes, such as amino sugar and carbohydrate metabolism or response to fungus, were also affected; however, they do not appear to be directly involved in defense against pathogenic bacteria.

In the case of Xe infection, an enrichment of DEGs associated with defense pathways and responses to stimuli was observed. Additionally, some of the DEGs identified following Xe infection were linked to secondary metabolic processes or cell wall metabolism. However, the number of genes involved was comparatively lower as compared to Xv, suggesting a reduced impact of these pathways under these conditions.

In addition to biological processes, GO functional analysis also revealed the molecular function (GO:MF) of the DEG-encoded products (Fig 7). This analysis showed that all DEGs common to both bacterial infections primarily encode chitinases, glucanases, expansins, and peroxidases. These enzymes are crucial for plant defense against fungal pathogens as they degrade chitin and β-glucans, major components of the fungal cell wall and are closely associated with plant immunity and the activation of defense responses. In Xe infected plants, all DEGs were also present following infection with Xv, except for chitinase CHI17 and glucan endo-1,3-β-glucosidase 3, which were downregulated and upregulated, respectively, only in response to the non-specific pathogen.

**Fig 7:**
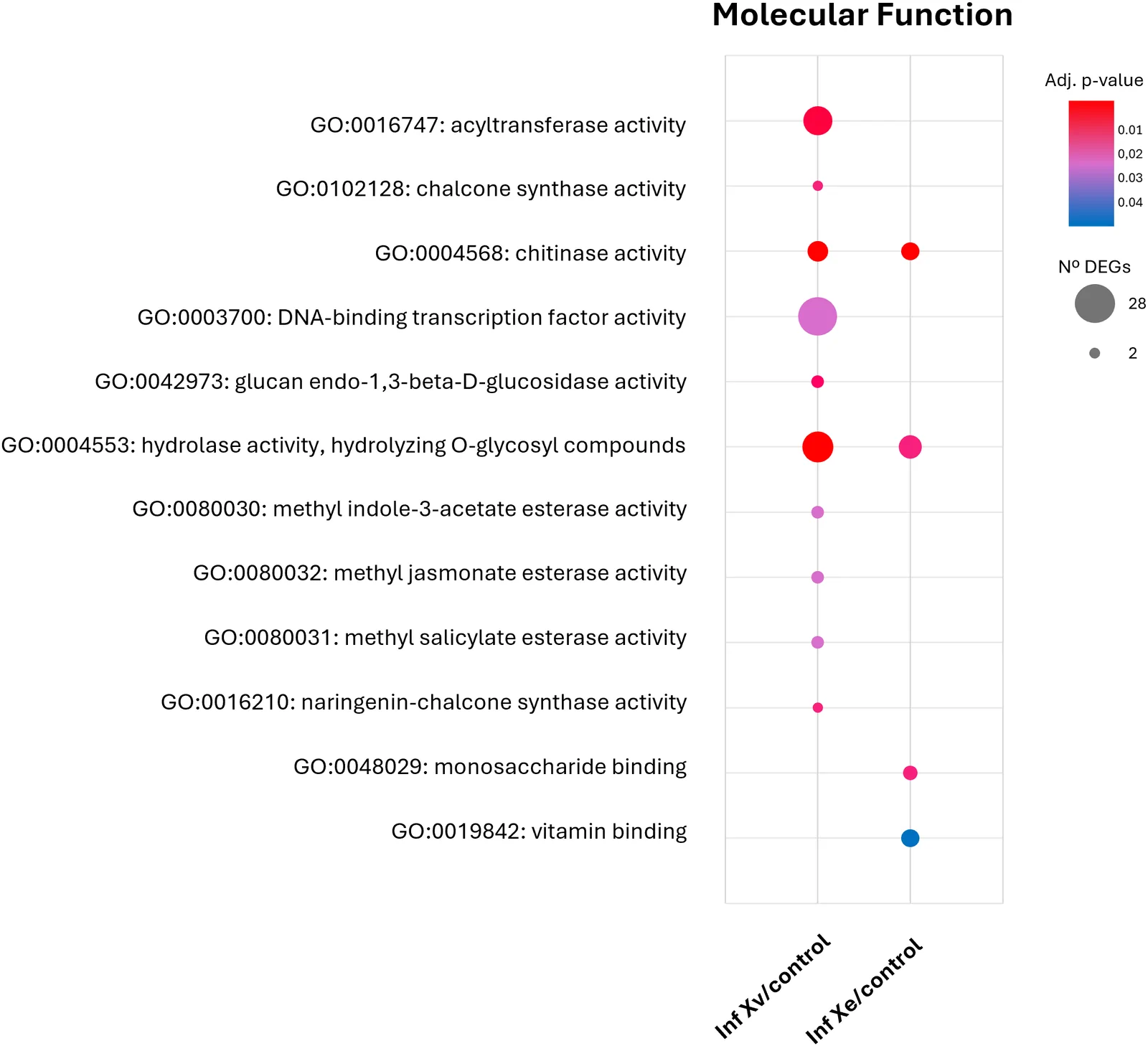
GO Molecular Function (GO:MF) classification reveals the specific activities of DEG products during Xv or Xe infection. Circle size represents the number of DEGs found in each comparison, while colour indicates significance.

Notably, there was an alteration of genes associated with the jasmonic acid, salicylic acid, chalcone synthase and indole-3-acetic acid pathways, which are well known for their roles in pathogenic bacteria stress responses, only in response to Xv.

### Common and differential genes in response to Xv and Xe infection in tomato plants

When examining the genes affected after infection by each pathogen, the activation of genes such as pathogenesis-related proteins (PR) including *PR1*, *PR2*, and *PR5*, was observed in both infections although it was markedly higher in Xv (Table 2). Likewise, a similar difference in the upregulation of genes associated with defense-related functions, such as FAD2-like, divinyl ether synthase (*DES*), and cytochrome P450, which are involved in the biosynthesis of fatty acids (Lee et al., 2020), antimicrobial compounds (Wasternack and Feussneret, 2018), and cell wall components (Pandian et al. 2020) were shown. Additionally, ethylene related genes 1-aminocyclopropane-1-carboxylate oxidase homolog 1 and *ACO4* were affected.

**Table 2:** Comparison of part of the common DEGs after infection with Xv or Xe.

Transcript ID	Description	INF Xv		INF Xe	
		FC	p.value	FC	p.value
XM_019214666	(-)-germacrene D synthase	19.48	3.98E-10	17.46	3.47E-15
XM_004245701	14 kDa proline-rich protein DC2.15-like	3.38	1.27E-05	4.40	8.21E-05
XM_004246449	1-aminocyclopropane-1-carboxylate oxidase homolog 1	8.52	1.46E-11	4.70	7.22E-05
XM_004248048	3-ketoacyl-CoA synthase 1	−3.30	1.85E-05	−2.13	3.77E-02
XM_004239907	abscisic acid receptor PYL4-like	9.30	3.14E-07	4.53	4.54E-03
XM_010318094	aldehyde dehydrogenase family 3 member F1	−2.31	1.59E-03	−2.23	3.01E-03
NM_001246916	aromatic amino acid decarboxylase 1A (AADC1A)	19.99	6.03E-17	6.49	3.20E-06
NM_001329206	aspartate protease family protein	5.64	1.28E-03	3.08	3.95E-04
NM_001247869	beta-1,3-glucanase	54.68	1.37E-06	6.07	1.11E-05
XM_004230589	calmodulin	10.14	1.24E-16	4.01	4.97E-04
XM_004236702	CASP-like protein PIMP1	6.76	1.37E-07	5.34	2.21E-04
NM_001247475	chitinase (CHI3)	5.75	1.41E-03	3.05	1.25E-03
XM_004249829	citrate-binding protein-like	16.18	8.41E-27	5.17	1.78E-07
XM_019213065	COBRA-like protein 4, transcript variant X2	10.00	2.27E-11	8.33	8.43E-08
XM_004251459	cysteine-rich repeat secretory protein 38	14.57	4.14E-22	6.56	9.21E-07
XM_004247228	cysteine-rich repeat secretory protein 55	7.60	4.67E-14	4.38	1.30E-05
XM_004243490	cytochrome b561 and DOMON domain-containing protein At5g47530	18.44	5.17E-19	4.36	2.53E-04
XM_004249066	cytochrome P450 98A3	55.98	4.25E-60	16.08	2.70E-20
XM_019211577	delta(12)-fatty-acid desaturase FAD2-like	69.70	3.21E-39	16.20	2.39E-13
XM_004248615	dirigent protein 22	22.24	1.95E-07	3.75	1.00E-07
NM_001247598	divinyl ether synthase (DES)	59.55	1.58E-43	14.05	5.89E-13
NM_001247498	EIX receptor 1 (Eix1)	16.26	3.24E-05	5.04	9.05E-04
XM_019214958	epidermis-specific secreted glycoprotein EP1	10.44	1.20E-11	4.47	3.78E-04
NM_001246999	ethylene-forming enzyme (ACO4)	8.74	3.54E-24	3.99	1.13E-06
NM_001247009	ethylene-responsive late embryogenesis-like protein (ER5)	−4.35	1.60E-03	−9.42	6.30E-05
NM_001247029	expansin (EXP1)	6.39	7.06E-11	3.15	1.94E-03
XM_004235585	GDSL esterase/lipase APG (LOC101252264)	−4.12	6.98E-07	−2.42	1.06E-02
NM_001312890	glucan endo-1,3-beta-glucosidase B	9.65	2.69E-26	4.42	4.11E-09
XM_004249032	lactosylceramide 4-alpha-galactosyltransferase	7.83	3.24E-07	5.46	4.61E-04
XM_004228389	leucine-rich repeat protein 1-like	28.88	9.24E-10	5.80	1.89E-05
XM_004250354	lignin-forming anionic peroxidase	5.15	9.43E-07	3.69	4.16E-04
XM_019213626	MDIS1-interacting receptor like kinase 2-like	5.21	6.83E-08	3.85	1.91E-04
NM_001320392	miraculin (Lemir)	36.44	1.45E-09	3.99	6.12E-04
NM_001247948	N-hydroxycinnamoyl-CoA:tyramine N-hydroxycinnamoyl transferase THT1–3 (THT1–3)	7.89	4.26E-05	3.01	8.77E-04
XM_010322044	patatin-like protein 2	19.61	5.98E-31	10.72	8.93E-13
NM_001247154	pathogenesis-related protein P2 (PR-P2)	47.67	6.76E-10	3.49	1.63E-04
NM_001323319	pathogenesis-related protein STH-2	55.08	2.55E-07	8.33	2.69E-07
NM_001247041	peroxidase (cevi16)	5.84	1.70E-04	4.07	7.85E-06
XM_004239916	peroxidase P7	10.30	1.42E-26	3.22	4.83E-05
NM_001247199	PR-1a1 protein (PR-1a1)	20.03	4.49E-20	8.49	1.51E-07
NM_001247232	PR-5x (PR-5)	45.20	2.22E-10	4.33	3.18E-03
XM_004244842	probable linoleate 9S-lipoxygenase 5	28.15	5.50E-07	8.45	1.30E-03
XM_004240049	probable polygalacturonase	−3.89	3.85E-04	−2.48	2.34E-02
NM_001316915	probable WRKY transcription factor 40	10.62	2.29E-13	4.94	2.79E-05
XM_004249973	protein DMR6-LIKE OXYGENASE 2-like	6.12	5.60E-09	5.14	1.69E-06
XM_004252797	protein ECERIFERUM 1	21.50	6.52E-23	9.50	1.49E-08
NM_001247686	proteinase inhibitor I (ER1)	5.61	7.12E-05	3.37	1.05E-04
XM_010315501	receptor-like protein 43	10.18	3.85E-07	9.27	1.20E-04
XM_010318787	serine/threonine-protein kinase D6PKL1	−2.47	7.50E-03	−2.11	4.63E-02
XM_004234090	suberization-associated anionic peroxidase 2-like	11.82	9.62E-11	3.92	3.28E-04
XM_019215389	subtilisin-like protease SBT1.7	30.83	2.85E-07	9.04	3.68E-04
XM_004235216	tropinone reductase homolog At5g06060-like, transcript variant X2	18.10	1.46E-15	8.53	2.43E-06
XM_004245236	tyramine N-feruloyltransferase 4/11-like	8.34	1.29E-19	5.37	9.11E-09
XR_003245086	U1 spliceosomal RNA	−3.45	1.02E-03	−3.59	6.61E-03
NM_001279343	WRKY transcription factor 81 (WRKY81)	13.00	3.65E-06	4.51	2.86E-06

Genes with a positive FC value relative to control plants are shown in yellow, while genes that are downregulated after infection are shown in blue (|FC| ≥ 2, p < 0.05). Complete data provided in S1 and S2 Files.

On the other hand, genes such as ethylene-responsive late embryogenesis-like protein (*ER5*) and GDSL esterase/lipase APG were downregulated in the presence of both pathogens. Moreover, U1 spliceosomal RNA, which is related to post-transcriptional regulatory mechanisms, was shown to be also downregulated. All common genes that were either repressed or induced as compared to the control plants remained consistently regulated in both systems, with no genes displaying opposite behavior between the two bacterial infections.

When focusing on genes induced exclusively following Xv infection (Table 3), genes encoding proteins commonly associated with defense, such as *PR4*, *WRKY75* or *Kunitz trypsin inhibitor 2-like*, were identified. It is also worth noticing *Pti5*, which is associated with the promotion of systemic acquired resistance, and *ACO1*, related to the hypersensitive response. Among the genes that were repressed after infection, a particularly notable decrease was observed in the expression of ribosomal RNAs.

**Table 3:** DEGs identified in tomato exclusively after infection with Xv compared to control plants.

Transcript ID	Description	FC	p.value
XM_004235444	kunitz trypsin inhibitor 2-like	87.10	4.77E-07
NM_001247594	pathogenesis-related protein P4 (P4)	22.90	2.02E-03
NM_001247058	Pti5	16.62	5.44E-05
NM_001323315	WRKY DNA binding protein 1 (WRKY75)	15.95	1.50E-05
NM_001247095	1-aminocyclopropane-1-carboxylate oxidase 1 (ACO1)	12.37	5.37E-04
XM_004235987	probable calcium-binding protein CML30	11.87	3.97E-03
XM_004249363	NDR1/HIN1-like protein 3, transcript variant X2	11.77	7.76E-05
NM_001247229	glucan endo-1,3-beta-D-glucosidase (Q`b)	10.74	2.07E-05
XM_004229987	sigma factor binding protein 1, chloroplastic	10.08	2.23E-04
XM_004249020	ammonium transporter 2	8.46	1.38E-02
XM_004246333	probable glutathione S-transferase	7.84	2.59E-18
NM_001329537	NAD(P)H:quinone oxidoreductase	5.87	5.72E-03
XM_004240685	oxalate--CoA ligase, transcript variant X2	5.85	3.00E-03
XM_004250542	leucine-rich repeat receptor-like tyrosine-protein kinase PXC3	5.73	1.60E-05
XM_004233819	LRR receptor-like serine/threonine-protein kinase FLS2	5.72	5.10E-06
XM_004237286	putative methyltransferase DDB_G0268948	5.47	5.95E-03
XM_004238068	cytochrome P450 81F3	5.38	1.14E-03
XM_004235262	mitochondrial phosphate carrier protein 3, mitochondrial	5.37	2.19E-08
XM_004247609	wound-induced proteinase inhibitor 1-like	5.34	7.87E-03
NM_001247020	phytophthora-inhibited protease 1 (pip1)	4.59	4.43E-02
NM_001247856	hexose transporter protein (ht2)	4.55	9.68E-10
NM_001247158	geranylgeranyl pyrophosphate synthase 1 (GGPS1)	4.41	9.40E-04
NM_001246911	2-oxoglutarate Fe(II) dependent oxygenase superfamily protein (SlDMR6-1)	4.30	5.78E-08
XM_004249751	BAG family molecular chaperone regulator 5, mitochondrial-like	4.15	1.13E-03
NM_001246943	mitochondrial small heat shock protein (MTSHP)	3.72	1.10E-03
XM_004232645	probable WRKY transcription factor 31, transcript variant X2	3.72	4.97E-05
NM_001320601	phenylalanine ammonia-lyase 2 (PAL2)	3.66	2.88E-08
XM_004232063	proline dehydrogenase 2, mitochondrial	3.54	5.48E-11
NM_001247928	formate dehydrogenase (fdh)	3.42	3.99E-08
NM_001328632	scopoletin glucosyltransferase (twi1)	3.37	1.61E-02
NM_001246993	1-aminocyclopropane-1-carboxylate synthase (ACS1A)	3.24	2.68E-07
NM_001246933	leucine aminopeptidase (LAPA1)	3.11	3.89E-02
NM_001247875	sucrose synthase (sus3)	3.07	3.92E-06
XM_004243473	endoglucanase 11	−3.42	9.72E-04
NM_001247104	chalcone synthase (CHS1)	−3.74	1.43E-05
XM_004237403	auxin-responsive protein SAUR68-like	−3.74	3.65E-04
XM_010318993	DExH-box ATP-dependent RNA helicase DExH17	−3.78	3.31E-06
XM_004245152	TATA-binding protein-associated factor 2N, transcript variant X1	−3.90	1.99E-03
XM_004247057	probable pectate lyase 8	−4.25	9.18E-04
XM_004250630	GDSL esterase/lipase At1g71691-like	−4.80	9.91E-04
rna-LyesC2t042	tRNA-Ile (trnI-GAU)	−6.00	1.83E-03
rna-NC_007898.3_132869–135678	23S ribosomal RNA	−8.96	7.25E-03
rna-LyesC2r002	23S ribosomal RNA	−12.19	7.77E-03
rna-LyesC2r001	16S ribosomal RNA	−14.67	1.99E-03
rna-NC_007898.3_137874–139373	16S ribosomal RNA	−14.81	2.26E-03

Only genes with higher positive and lower negative FC values relative to control plants are shown (|FC| ≥ 2, p < 0.05). Complete data in S1 File.

On the other hand, regarding the DEGs affected following Xe infection (Table 4), as previously mentioned, an overall reduction in fold change values was observed, indicating lower levels of gene induction and repression compared to Xv infection. Furthermore, many of these genes are poorly characterized in existing databases, limiting the available functional information. Nevertheless, some upregulated genes involved in RNA modification (XM_019212051; Pentatricopeptide repeat-containing protein, PPR) and protein ubiquitination (XM_004240128; U-box E3 ubiquitine ligase) were identified. In contrast, certain genes associated with defense and stimuli response were clearly repressed, such as ethylene responsive transcription factors or glutathione S-transferase (*GST-L3*), which is implicated in the modulation of the ROS levels after the pathogen attack.

**Table 4:** DEGs identified in tomato exclusively after infection with Xe compared to control plants.

Transcript ID	Description	FC	p.value
XM_019212051	pentatricopeptide repeat-containing protein At4g18840	11.05	2.69E-03
XM_004240128	U-box domain-containing protein 26-like	4.89	3.01E-02
XM_004242116	HIPL1 protein	4.22	3.91E-03
XM_026032059	TMV resistance protein N-like	3.10	5.43E-03
XM_004249376	calcium homeostasis endoplasmic reticulum protein, transcript variant X3	2.95	9.54E-03
XM_004236892	glucan endo-1,3-beta-glucosidase 3	2.87	7.28E-03
XM_010322158	catalase isozyme 3-like	2.86	1.87E-02
XM_004235244	SWI/SNF complex subunit SWI3A	2.83	2.40E-02
XM_004248669	glycine-rich RNA-binding protein	2.76	1.61E-02
XM_010314951	DDT domain-containing protein PTM	2.62	1.64E-02
XM_004246782	wall-associated receptor kinase 2-like	2.61	1.67E-02
XM_010321784	exocyst complex component SEC5A, transcript variant X1	2.60	4.50E-03
XM_004248126	putative late blight resistance protein homolog R1B-12, transcript variant X4	2.59	1.31E-02
XM_004248035	aspartyl protease family protein 2	2.58	3.35E-03
XM_004243438	probable WRKY transcription factor 31	2.54	3.39E-02
XM_004240144	nuclear pore complex protein NUP1-like	2.43	1.17E-02
XM_004232844	VQ motif-containing protein 4	2.42	1.98E-02
XM_004239610	methyltransferase-like protein 1, transcript variant X2	2.41	2.23E-02
XM_004250288	phosphatidylinositol 4-phosphate 5-kinase 2	2.41	1.26E-02
XM_010323012	lysM domain receptor-like kinase 3	2.36	3.17E-02
XM_004247326	probable LRR receptor-like serine/threonine-protein kinase At1g67720	2.36	1.83E-02
XM_004250777	pre-mRNA-processing protein 40A, transcript variant X1	2.34	5.64E-03
XM_004246565	E3 ubiquitin-protein ligase RFWD3	2.33	4.33E-02
XM_004244754	potassium channel KAT3	2.30	3.00E-02
XM_004230910	transcription termination factor MTEF1, chloroplastic	2.22	6.82E-03
NM_001247778	dehydration-responsive element binding protein 3 (DREB3)	2.12	1.21E-02
XM_010322827	GTP-binding protein OBGC, chloroplastic, transcript variant X3	2.06	4.16E-02
XM_004230755	CDPK-related kinase 5	2.05	1.25E-02
XM_004246664	glutathione S-transferase L3, transcript variant X1	−2.11	5.54E-03
XM_004252032	zeatin O-glucosyltransferase-like	−2.27	3.74E-02
XM_004229765	glutathione S-transferase zeta class-like, transcript variant X1	−2.48	1.76E-03
NM_001247471	chitinase (CHI17)	−2.56	5.15E-04
NM_001247823	gibberellin 2-oxidase (GA2ox4)	−2.64	4.18E-02
NM_001328505	proteinase inhibitor I	−2.68	4.13E-03
XM_004238707	thioredoxin H4-like	−2.88	5.42E-03
XM_019215208	subtilisin-like protease SBT1.2	−2.94	2.34E-02
XM_004229716	ethylene-responsive transcription factor ERF038-like	−3.10	2.81E-02
NR_137325	U1 small nuclear RNA (U1.8)	−3.13	6.50E-04
XM_004233761	berberine bridge enzyme-like 22	−3.53	2.28E-02
XM_004235388	light-harvesting complex-like protein 3 isotype 2, chloroplastic, transcript variant X2	−3.71	1.51E-02
NR_137324	U1 small nuclear RNA (U1.7)	−3.77	4.87E-04
XM_004245360	non-specific lipid-transfer protein 1-like	−3.90	1.39E-03
XM_004237405	auxin-responsive protein SAUR68-like	−3.92	1.20E-02
XR_003244172	U1 spliceosomal RNA	−3.97	1.92E-03
XM_019211293	persulfide dioxygenase ETHE1 homolog, mitochondrial-like	−4.61	3.74E-03
XM_004245472	ethylene-responsive transcription factor ERF061	−4.63	4.10E-07
XM_004248515	hyoscyamine 6-dioxygenase-like	−4.99	6.36E-03
XM_004243804	mitogen-activated protein kinase kinase kinase 18-like	−5.51	3.67E-04
XM_004244583	ethylene-responsive transcription factor ERF061	−5.55	2.04E-04
XR_003246304	U4 spliceosomal RNA	−10.83	4.03E-03

Only genes with higher positive and lower negative FC values relative to control plants are shown (|FC| ≥ 2, p < 0.05). Complete data in S2 File.

## Discussion

*Xanthomonas* spp. comprises a diverse and complex group of plant-associated bacteria, many of which are pathogenic and cause economically significant diseases across a wide range of crops [13]. In this context, we conducted a comprehensive transcriptomic analysis to elucidate the molecular mechanisms underlying differences in infection patterns between two strains, Xv and Xe, belonging to distinct *Xanthomonas* spp., originally isolated from tomato and pepper, respectively, yet both capable of infecting tomato plants. This experimental design enables a direct comparison of tomato responses to both strains under controlled conditions, with tomato serving as a common host for two distinct bacterial species associated with bacterial spot disease but differing in host origin. Collectively, this approach provides a foundation for further characterization of host-*Xanthomonas* interactions.

Although throughout the 20th century both bacteria were considered a single species, phylogenetic studies have since led to significant reclassifications [7]. These changes were primarily driven by differences in genomic content, particularly in metabolic pathways and virulence associated genes, which may play a crucial role in determining their specificity for colonizing different plant species. Despite their genetic similarity, these bacteria induce slightly different effects on the plant, highlighting variations in their pathogenic interactions, infection dynamics and plant’s response.

Our results showed that Xv and Xe were able to colonize the leaf tissue after inoculation, indicating their capacity to establish infection in the host plant. Nevertheless, even when a higher inoculum was applied, Xe strain exhibited lower spread during the infection process compared to Xv strain at 5 days post infection*.* The difference in symptom severity could be attributed to multiple factors, including variations in the virulence mechanisms employed by each bacterium, previously reported differences in their ability to suppress or evade plant immune responses, and distinct host adaptation strategies [5]. Additionally, it is possible that Xe strain triggered a more efficient defense response in tomato, thereby limiting disease progression. Understanding these differences at the molecular level could provide valuable insights into host-pathogen interactions and the determinants of bacterial pathogenicity in tomato plants.

In the correlation analysis based on transcriptomics data, control samples and those infected with Xe clustered more closely together, whereas samples infected with Xv grouped separately. These findings suggest that infection with Xv elicited a stronger transcriptional reprogramming in tomato than infection with Xe, under the conditions tested. This observation was further supported by the distinct number of DEGs identified in response to each bacterium. Xv infection not only caused more severe tissue damage, but also elicited a broader transcriptomic reprogramming, with more genes both upregulated and downregulated compared to control plants. Notably, these DEGs also exhibited higher fold change magnitudes, supporting a stronger transcriptional response.

To elucidate potential pathways affected after infection, DEGs were classified based on homology into genes encoding proteins involved in different biological and molecular processes. Both pathogens induced significant changes in pathways linked to cell wall metabolism and phenylpropanoid biosynthesis. This is particularly relevant given the plant cell wall serves as the first line of defense against pathogen invasion, acting not only as a physical barrier to prevent pathogen entry but also as an active participant in defense by detecting pathogen-associated molecular patterns (PAMPs) and triggering immune responses [29]. Moreover, considered with these defense related changes, altered chitinase activity was also observed; these enzymes cooperate with glucanases in plant defense mechanisms, contributing either through degradation of microbial exopolysaccharides or activating defense-related signaling cascades [30].

Examining the differential host response between the two bacterial infections, plants infected with Xv showed an enrichment of genes related to ornithine metabolism. Ornithine is a precursor molecule of polyamines, which play a crucial role in stress response by modulating the oxidative state as a defense mechanism against pathogens [31]. Moreover, genes related to salicylic acid metabolism, a key hormone in the plant’s innate immunity [32], were also enriched in infected plants with this bacterium. Salicylic acid triggers both local and systemic responses to biotic stress. Previous studies have reported that the chalcone synthase (CHS) expression leads to the accumulation of flavonoid and isoflavonoid phytoalexins and contributes to the salicylic acid defense pathway [33]. Its repression during Xv infection suggests that the bacterium may actively suppress the plant’s defense mechanisms to enhance its survival and establish a successful infection. In the case of Xe, interestingly, specific DEGs observed after the infection were associated with general plant defense pathways and responses to stimuli.

These differential responses observed suggest that the tomato plant employs distinct defense strategies against each bacterial strain. The observed mechanisms ranged from responses potentially limiting bacterial growth to the activation of specialized immune pathway responses and the reinforcement of structural barriers, indicating the plant’s ability to recognize and mount appropriate responses that may be influenced by each pathogen’s infection strategy and host interaction mode.

Following the analysis of GO terms associated with altered biological processes and molecular functions, we next focused on identifying key genes involved in tomato plants’s interaction with both *Xanthomonas* strains. Specifically, we examined genes that were differentially expressed in response to both pathogens to determine their potential role in plant’s defense mechanisms. Tomato plants exhibited a conserved immune response involving genes related to basal and systemic defenses, including chitinases, peroxidases and glucanases. Additionally, the induction of various genes encoding PR proteins was observed, specifically *PR1*, *PR2*, and *PR5*. Together, the upregulation of these genes following infection with both bacteria suggests the activation of salicylic acid-based defenses, promoting an immune response that may help to restrict bacterial colonization [34]. Upregulation of genes associated with ethylene signaling pathway (*1-aminocyclopropane-1-carboxylate oxidase homolog 1* and *ACO4*) and cell wall modification (Expansins, COBRA-like) was also detected [35–37]. These findings indicate that tomato displays a robust and conserved defense response regardless of the *Xanthomonas* strain’s origin. However, higher expression levels were found during Xv strain infection indicating that this strain elicited a stronger transcriptional response under the experimental conditions tested.

Among the commonly downregulated DEGs, we identified the ethylene-responsive late embryogenesis-like protein (ER5) and the GDSL esterase/lipase APG. Interestingly, both genes are associated with ethylene-mediated response to abiotic and biotic stress [38–40]. The stronger repression of ER5 in Xe infection compared to Xv may be linked to the presence of the effector XopD in this strain (results not shown), which has previously been reported to suppress ethylene production in the host to mitigate ethylene-induced immune response upon infection [41]. Additionally, we also observed a downregulation of the gene encoding for the U1 spliceosomal RNA. Given the crucial role of alternative splicing (AS) regulation in plant immunity, it is possible that pathogens have evolved effectors to reprogram host AS by targeting the spliceosome, although this remains unclear [42].

Focusing on the infection with Xv, we identified genes encoding proteins typically also associated with plant defense, such as *PR4* or *WRKY75*, which may play a more specific role in the plant’s response to this bacterium. Additionally, we identified other genes, such as the *Kunitz trypsin inhibitor 2-like* (*KNTZ*), which had been previously linked to the inhibition of proteases like trypsin and chymotrypsin, and playing an important role in plant resistance to insects or nematodes. Interestingly, Tortosa et al. [43] reported an increase in *KNTZ*s and their correlation with *PR*s type 6, suggesting a potential key role in *Brassica oleracea* defense response to *Xanthomonas campestris* pv *campestris*. Nevertheless, given that *PR4* and *KNTZ* are typically associated with fungal and insect stress responses, we cannot rule out the possibility that the bacteria could be manipulating the plant to activate alternative defensive pathways that would not compromise its own spread.

Notably, among the genes repressed after infection with Xv, we found a decrease in the expression of ribosomal RNAs, both 16S and 23S. This reduction may be part of the plant’s strategy to redirect resources towards defense mechanisms rather than routine cellular processes such as protein synthesis. Consequently, the response to Xv strain was actively focused on defense, suggesting that the plant perceived the bacterium as a threat and triggered a robust immune response. However, Xv infection resulted in extensive disease development, suggesting that the induced responses were insufficient to restrict pathogen progression.

In the case of the DEGs observed only after Xe infection, it is noteworthy that the expression levels were generally much lower in general, both for upregulated and downregulated genes. Furthermore, the response to Xe strain appeared to be also more metabolically oriented. There was a reduced activation of specific defense-related genes, while genes associated with calcium homeostasis, secretion, and general stress resistance were more prevalent.

Among the most induced genes after Xe infection, we identified genes related in the database to RNA modification and protein ubiquitination. However, these genes seem to be more associated with a general response to biotic and abiotic stresses [44,45]. Remarkably, the *PPR* gene showed a significant increase in expression. These proteins act at the post-transcriptional level both in mitochondria and chloroplasts, playing a role in the plant survival, particularly during growth and development under abiotic and biotic stress [46]. For instance, PPR proteins appear to be also important during biotic stress responses against necrotrophs like *Botrytis cinerea* or hemibiotrophs like *Pseudomonas syringae* [47]. On the other hand, U-box E3 ubiquitin ligase is involved in hormonal pathways and biotic stress [48], being linked, among others, to the activation of the immune response dependent on PRRs, which are key components of the innate immune system responsible for detecting PAMPs [49].

Therefore, the observed interaction between the tomato plant and Xv could be explained as an attempt by the plant to halt the attack once the infection has been detected, leading to the high expression levels of these proteins that have clearly been reported as in response to biotrophic pathogens. However, given the severity of the lesions and the ability of Xv to proliferate within the plant, it is evident that the plant was not able to mount an effective defense, either due to failure to detect at early stages, resulting in a delayed response, or due to the bacterium’s capacity to modulate the host immunity by redirecting canonical defense pathways toward less specific responses, such as those associated with fungal or insect attack. In contrast, the Xe strain isolated from pepper required a higher inoculum to infect tomato, and the transcriptomic profile observed after infection differs from that of Xv. This could also be explained as an inability of Xe to deceive the plant, leading to a slower and less severe infection that does not necessitate a major reconfiguration of the plant’s metabolism. Nevertheless, it is important to note that the observed differences between bacterial strains should be interpreted in light of the temporal dynamics of the host response. Therefore, timing remains an unexplored but potentially important factor that could partially explain the differences observed between strains and should be addressed in future time-course analyses.

Comparative genomic and functional analyses of the bacterial spot complex have revealed that Xv and Xe harbor distinct yet overlapping repertoires of T3SS, whose dynamic evolution contributes to host specificity and pathogenic diversity [5,50]. The genomes of the two bacterial strains used in this study have been sequenced and deposited in GenBank, effectors of the T3SS are known (BioSample accessions SAMN53467229, SAMN53467230) and both possess distinct and diverse sets of them. Those effectors have specific functions and localizes in different regions of the plant cell, participating in various biological processes and some have been described as suppressors of plant defense responses. Among the effectors that distinguish the Xv strain from the Xe strain, XopJ2 (syn AvrBsT) of the XopJ class [3,25] is specifically present in Xv. AvrBsT significantly compromised callose deposition and the expression of defense-marker genes in tomato plants when inoculated at high bacterial titers. Studies also showed that genes of XopJ class reduced salicylic acid levels, causing changes in gene expression associated with defense and senescence [51,52]. However, to date, there is no experimental evidence demonstrating a direct role for XopJ2 in the regulation of systemic acquired resistance (SAR) or long-distance defense signaling; therefore, such functional roles remain speculative and cannot be directly extrapolated to XopJ2 in the absence of dedicated experimental validation. Meanwhile, Xe presents an even broader pool of effectors, which include AvrBs3, extensively studied due to its role in tomato resistance to infection by this bacterium, as it triggers specific immune responses that limit pathogen progression [15,53]. Notably, the different effector repertoire in both strains appears to correlate with the induction of a more widespread transcriptional response in Xv compared to Xe.

In summary, Xv and Xe represent two closely related yet distinct species within the bacterial spot pathogen complex, each with unique genetic, biochemical, and pathogenic profiles. Our transcriptomic analysis further revealed both shared and species-specific patterns of gene expression, highlighting differences in the molecular responses of these pathogens under the conditions examined. The elucidation of these differences has important implications for disease diagnostics, resistance breeding, and management strategies. As the bacterial spot disease continues to pose a threat to global food security, further research is needed to explore the molecular mechanisms underlying host-pathogen interactions, the genetic basis of host specificity, and the development of more effective disease control measures.

## Supporting information

S1 File List of differentially expressed transcripts in Xv-infected plants compared to control plants (|FC| ≥ 2, p < 0.05). The file includes normalized expression values, statistical significance measures, and Gene Ontology (GO) classifications based on molecular function (GO:MF), biological process (GO:BP), and cellular component (GO:CC).
(XLSX)

S2 File List of differentially expressed transcripts in Xe-infected plants compared to control plants (|FC| ≥ 2, p < 0.05). The file includes normalized expression values, statistical significance measures, and Gene Ontology (GO) classifications based on molecular function (GO:MF), biological process (GO:BP), and cellular component (GO:CC).
(XLSX)

S1 Table Slope, coefficient of determination (R^2^) and amplification efficiency of each primer pair used in the RT-qPCR. Amplification efficiency was calculated form the slope of each standard curve.
(DOCX)

S2 Table Summary of raw sequencing data metrics. Total Bases: total raw sequencing data volume (reads × read length). Total Reads: total number of sequencing fragments generated. GC Content (%): proportion of guanine (G) and cytosine (C) bases in the quality-filtered data. Q20 (%) and Q30 (%): percentages of bases with Phred quality scores ≥ 20 and ≥ 30, respectively.
(DOCX)

S3 Table Summary of Clean sequencing data quality metrics. Total Bases: total sequencing data volume post-QC (clean reads × read length). Total Reads: total number of high-quality reads after filtering (adapter trimming and low-quality read removal). GC ratio (%): proportion of guanine (G) and cytosine (C) bases. Q20 (%) and Q30 (%): percentages of bases with Phred quality scores ≥ 20 and ≥ 30, respectively.
(DOCX)

S4 Table Summary of sequence mapping statistics. Processed Reads: total number of reads analyzed after quality control. Mapped Reads: number of reads successfully aligned to the reference genome. Mapping Ratio: percentage of reads mapped to reference. Unmapped Reads: number of reads failing to align. Unmapped Ratio: percentage of reads not aligned to the reference.
(DOCX)

## References

[1] Potnis N, Colee J, Jones JB, Barak JD. Plant pathogen-induced water-soaking promotes *Salmonella enterica* growth on tomato leaves. Appl Environ Microbiol. 2015;81(23):8126–34. doi: 10.1128/AEM.01926-15 26386057 PMC4651078

[2] Osdaghi E, Jones JB, Sharma A, Goss EM, Abrahamian P, Newberry EA, et al. A centenary for bacterial spot of tomato and pepper. Mol Plant Pathol. 2021;22(12):1500–19. doi: 10.1111/mpp.13125 34472193 PMC8578828

[3] Costa J, Pothier J, Bosis E, Boch J, Kölliker R, Koebnik R. A community-curated DokuWiki resource on diagnostics, diversity, pathogenicity, and genetic control of Xanthomonads. Mol Plant-Microbe Interact. 2024;37(3):347–53. doi: 10.1094/MPMI-11-23-0184-FIA 38114082

[4] Schwartz AR, Potnis N, Timilsina S, Wilson M, Patané J, Martins J Jr, et al. Phylogenomics of Xanthomonas field strains infecting pepper and tomato reveals diversity in effector repertoires and identifies determinants of host specificity. Front Microbiol. 2015;6:535. doi: 10.3389/fmicb.2015.00535 26089818 PMC4452888

[5] Potnis N, Timilsina S, Strayer A, Shantharaj D, Barak JD, Paret ML, et al. Bacterial spot of tomato and pepper: Diverse Xanthomonas species with a wide variety of virulence factors posing a worldwide challenge. Mol Plant Pathol. 2015;16(9):907–20. doi: 10.1111/mpp.12244 25649754 PMC6638463

[6] Timilsina S, Potnis N, Newberry EA, Liyanapathiranage P, Iruegas-Bocardo F, White FF, et al. Xanthomonas diversity, virulence and plant-pathogen interactions. Nat Rev Microbiol. 2020;18(8):415–27. doi: 10.1038/s41579-020-0361-8 32346148

[7] Jones JB, Lacy GH, Bouzar H, Stall RE, Schaad NW. Reclassification of the xanthomonads associated with bacterial spot disease of tomato and pepper. Syst Appl Microbiol. 2004;27(6):755–62. doi: 10.1078/0723202042369884 15612634

[8] Dhakal U, Dobhal S, Alvarez AM, Arif M. Phylogenetic analyses of Xanthomonads causing bacterial leaf spot of tomato and pepper: *Xanthomonas euvesicatoria* revealed homologous populations despite distant geographical distribution. Microorganisms. 2019;7(10):462. doi: 10.3390/microorganisms7100462 31623235 PMC6843189

[9] Potnis N. Harnessing eco-evolutionary dynamics of Xanthomonads on tomato and pepper to tackle new problems of an old disease. Annu Rev Phytopathol. 2021;59:289–310. doi: 10.1146/annurev-phyto-020620-101612 34030449

[10] Roach R, Mann R, Gambley CG, Chapman T, Shivas RG, Rodoni B. Genomic sequence analysis reveals diversity of Australian Xanthomonas species associated with bacterial leaf spot of tomato, capsicum and chilli. BMC Genom. 2019;20(1):310. doi: 10.1186/s12864-019-5600-x 31014247 PMC6480910

[11] Burlakoti RR, Hsu C-F, Chen J-R, Wang J-F. Population dynamics of Xanthomonads associated with bacterial spot of tomato and pepper during 27 years across Taiwan. Plant Dis. 2018;102(7):1348–56. doi: 10.1094/PDIS-04-17-0465-RE 30673574

[12] Potnis N, Krasileva K, Chow V, Almeida NF, Patil PB, Ryan RP, et al. Comparative genomics reveals diversity among Xanthomonads infecting tomato and pepper. BMC Genom. 2011;12:146. doi: 10.1186/1471-2164-12-146 21396108 PMC3071791

[13] Jacques M-A, Arlat M, Boulanger A, Boureau T, Carrère S, Cesbron S, et al. Using ecology, physiology, and genomics to understand host specificity in Xanthomonas. Annu Rev Phytopathol. 2016;54:163–87. doi: 10.1146/annurev-phyto-080615-100147 27296145

[14] Felipe V, Romero AM, Montecchia MS, Vojnov AA, Bianco MI, Yaryura PM. *Xanthomonas vesicatoria* virulence factors involved in early stages of bacterial spot development in tomato. Plant Pathol. 2018;67(9):1936–43. doi: 10.1111/ppa.12905

[15] Bianco MI, Ponso MA, Garita-Cambronero J, Conforte VP, Galván TE, Dunger G, et al. Genomic and phenotypic insight into *Xanthomonas vesicatoria* strains with different aggressiveness on tomato. Front Microbiol. 2023;14:1185368. doi: 10.3389/fmicb.2023.1185368 37440880 PMC10333488

[16] Preangtong Y, Patarapuwadol S, Phiriyangkul P, Kanhayart T, Kositcharoenkul N, Kositratana W, et al. Characterisation and genomic diversity of Xanthomonas species causing bacterial spot disease of tomato and pepper in Thailand. Plant Pathol. 2025;74(5):1315–34. doi: 10.1111/ppa.14094

[17] Bouzar H, Jones J, Stall R, Hodge N, Minsavage G, Benedict A, et al. Physiological, chemical, serological, and pathogenic analyses of a worldwide collection of *Xanthomonas campestris* pv. *vesicatoria* strains. Phytopathology. 1994;84(7):663–71. doi: 10.1094/Phyto-84-663

[18] Kizheva Y, Vancheva T, Hristova P, Stoyanova M, Stojanovska P, Moncheva P, et al. Identification of Xanthomonas strains from tomato and pepper and their sensitivity to antibiotics and copper. Bulg J Agri Sci. 2013;19(2):80–2.

[19] Chan JW, Goodwin PH. The molecular genetics of virulence of *Xanthomonas campestris*. Biotechnol Adv. 1999;17(6):489–508. doi: 10.1016/s0734-9750(99)00025-7 14538126

[20] Jones JDG, Dangl JL. The plant immune system. Nature. 2006;444(7117):323–9. doi: 10.1038/nature05286 17108957

[21] Aerts N, Chhillar H, Ding P, Van Wees SCM. Transcriptional regulation of plant innate immunity. Essays Biochem. 2022;66(5):607–20. doi: 10.1042/EBC20210100 35726519 PMC9528082

[22] Kakkar A, Nizampatnam NR, Kondreddy A, Pradhan BB, Chatterjee S. *Xanthomonas campestris* cell-cell signalling molecule DSF (diffusible signal factor) elicits innate immunity in plants and is suppressed by the exopolysaccharide xanthan. J Exp Bot. 2015;66(21):6697–714. doi: 10.1093/jxb/erv377 26248667 PMC4623683

[23] Yu ZH, Wang JF, Stall RE, Vallejos CE. Genomic localization of tomato genes that control a hypersensitive reaction to *Xanthomonas campestris* pv. *vesicatoria* (Doidge) dye. Genetics. 1995;141(2):675–82. doi: 10.1093/genetics/141.2.675 8647402 PMC1206765

[24] Vasileva K, Bogatzevska N. Statistical analysis of resistance variability of pepper accessions to *Xanthomonas euvesicatoria* and *Xanthomonas vesicatoria*. Agri Sci Tech. 2021;13:339–43. doi: 10.15547/ast.2021.04.055

[25] Kim NH, Choi HW, Hwang BK. *Xanthomonas campestris* pv. *vesicatoria* effector AvrBsT induces cell death in pepper, but suppresses defense responses in tomato. Mol Plant Microbe Interact. 2010;23(8):1069–82. doi: 10.1094/MPMI-23-8-1069 20615117

[26] Timilsina S, Jibrin MO, Potnis N, Minsavage GV, Kebede M, Schwartz A, et al. Multilocus sequence analysis of xanthomonads causing bacterial spot of tomato and pepper plants reveals strains generated by recombination among species and recent global spread of *Xanthomonas gardneri*. Appl Environ Microbiol. 2015;81(4):1520–9. doi: 10.1128/AEM.03000-14 25527544 PMC4309686

[27] Hoagland D, Arnon D. The water-culture method for growing plants without soil. Calif. Agric. Exp. Stn. Circ. 1950;347:32.

[28] Bolger AM, Lohse M, Usadel B. Trimmomatic: A flexible trimmer for Illumina sequence data. Bioinformatics. 2014;30(15):2114–20. doi: 10.1093/bioinformatics/btu170 24695404 PMC4103590

[29] Underwood W. The plant cell wall: A dynamic barrier against pathogen invasion. Front Plant Sci. 2012;3. doi: 10.3389/fpls.2012.00085 PMC3355688 22639669

[30] Vaghela B, Vashi R, Rajput K, Joshi R. Plant chitinases and their role in plant defense: A comprehensive review. Enzyme Microb Technol. 2022;159:110055. doi: 10.1016/j.enzmictec.2022.110055 35537378

[31] González-Hernández AI, Scalschi L, Troncho P, García-Agustín P, Camañes G. Putrescine biosynthetic pathways modulate root growth differently in tomato seedlings grown under different N sources. J Plant Physiol. 2022;268:153560. doi: 10.1016/j.jplph.2021.153560 34798464

[32] Vlot AC, Dempsey DA, Klessig DF. Salicylic Acid, a multifaceted hormone to combat disease. Annu Rev Phytopathol. 2009;47:177–206. doi: 10.1146/annurev.phyto.050908.135202 19400653

[33] Dao TTH, Linthorst HJM, Verpoorte R. Chalcone synthase and its functions in plant resistance. Phytochem Rev. 2011;10(3):397–412. doi: 10.1007/s11101-011-9211-7 21909286 PMC3148432

[34] van Loon LC, Rep M, Pieterse CMJ. Significance of inducible defense-related proteins in infected plants. Annu Rev Phytopathol. 2006;44:135–62. doi: 10.1146/annurev.phyto.44.070505.143425 16602946

[35] Barry CS, Blume B, Bouzayen M, Cooper W, Hamilton AJ, Grierson D. Differential expression of the 1-aminocyclopropane-1-carboxylate oxidase gene family of tomato. Plant J. 1996;9(4):525–35. doi: 10.1046/j.1365-313x.1996.09040525.x 8624515

[36] Cao Y, Tang X, Giovannoni J, Xiao F, Liu Y. Functional characterization of a tomato COBRA-like gene functioning in fruit development and ripening. BMC Plant Biol. 2012;12:211. doi: 10.1186/1471-2229-12-211 23140186 PMC3533923

[37] Tancos MA, Lowe-Power TM, Peritore-Galve FC, Tran TM, Allen C, Smart CD. Plant-like bacterial expansins play contrasting roles in two tomato vascular pathogens. Mol Plant Pathol. 2018;19(5):1210–21. doi: 10.1111/mpp.12611 28868644 PMC5835177

[38] Zegzouti H, Jones B, Marty C, Lelièvre JM, Latché A, Pech JC, et al. ER5, a tomato cDNA encoding an ethylene-responsive LEA-like protein: Characterization and expression in response to drought, ABA and wounding. Plant Mol Biol. 1997;35(6):847–54. doi: 10.1023/a:1005860302313 9426604

[39] Müller M, Munné-Bosch S. Ethylene response factors: A key regulatory hub in hormone and stress signal. Plant Physiol. 2015;169(1):32–41. doi: 10.1104/pp.15.00677 PMC4577411 26103991

[40] Shen G, Sun W, Chen Z, Shi L, Hong J, Shi J. Plant GDSL esterases/lipases: Evolutionary, physiological and molecular functions in plant development. Plants (Basel). 2022;11(4):468. doi: 10.3390/plants11040468 PMC8880598 35214802

[41] Nagabhyru P, Dinkins RD, Wood CL, Bacon CW, Schardl CL, et al. Tall fescue endophyte effects on tolerance to water-deficit stress. BMC Plant Biol. 2013;13:127. doi: 10.1186/1471-2229-13-127 24015904 PMC3848598

[42] Yang S, Tang F, Zhu H. Alternative splicing in plant immunity. Int J Mol Sci. 2014;15(6):10424–45. doi: 10.3390/ijms150610424 24918296 PMC4100160

[43] Tortosa M, Velasco P, Rodríguez VM, Cartea ME. Changes in Brassica oleracea leaves infected with *Xanthomonas campestris* pv. *campestris* by proteomics analysis. Front Plant Sci. 2022;12:781984. doi: 10.3389/fpls.2021.781984 35211128 PMC8860909

[44] Ge L, Pan F, Jia M, Pott DM, He H, Shan H, et al. RNA modifications in plant biotic interactions. Plant Commun. 2025;6(2):101232. doi: 10.1016/j.xplc.2024.101232 39722456 PMC11897454

[45] Langin G, González-Fuente M, Üstün S. The plant ubiquitin-proteasome system as a target for microbial manipulation. Annu Rev Phytopathol. 2023;61:351–75. doi: 10.1146/annurev-phyto-021622-110443 37253695

[46] Wang Y, Tan B-C. Pentatricopeptide repeat proteins in plants: Cellular functions, action mechanisms, and potential applications. Plant Commun. 2025;6(2):101203. doi: 10.1016/j.xplc.2024.101203 39644091 PMC11897456

[47] Park YJ, Lee HJ, Kwak KJ, Lee K, Hong SW, Kang H. MicroRNA400-guided cleavage of Pentatricopeptide repeat protein mRNAs Renders Arabidopsis thaliana more susceptible to pathogenic bacteria and fungi. Plant Cell Physiol. 2014;55(9):1660–8. doi: 10.1093/pcp/pcu096 25008976

[48] Sharma B, Taganna J. Genome-wide analysis of the U-box E3 ubiquitin ligase enzyme gene family in tomato. Sci Rep. 2020;10(1):9581. doi: 10.1038/s41598-020-66553-1 32533036 PMC7293263

[49] Lu D, Lin W, Gao X, Wu S, Cheng C, Avila J, et al. Direct ubiquitination of pattern recognition receptor FLS2 attenuates plant innate immunity. Science. 2011;332(6036):1439–42. doi: 10.1126/science.1204903 21680842 PMC3243913

[50] White FF, Potnis N, Jones JB, Koebnik R. The type III effectors of Xanthomonas. Mol Plant Pathol. 2009;10(6):749–66. doi: 10.1111/j.1364-3703.2009.00590.x 19849782 PMC6640274

[51] Bartetzko V, Sonnewald S, Vogel F, Hartner K, Stadler R, Hammes UZ, et al. The *Xanthomonas campestris* pv. *vesicatoria* type III effector protein XopJ inhibits protein secretion: Evidence for interference with cell wall-associated defense responses. Mol Plant Microbe Interact. 2009;22(6):655–64. doi: 10.1094/MPMI-22-6-0655 19445590

[52] Üstün S, Bartetzko V, Börnke F. The *Xanthomonas campestris* type III effector XopJ targets the host cell proteasome to suppress salicylic-acid mediated plant defence. PLoS Pathog. 2013;9(6):e1003427. doi: 10.1371/journal.ppat.1003427 23785289 PMC3681735

[53] Gürlebeck D, Thieme F, Bonas U. Type III effector proteins from the plant pathogen Xanthomonas and their role in the interaction with the host plant. J Plant Physiol. 2006;163(3):233–55. doi: 10.1016/j.jplph.2005.11.011 16386329

