# Supplementary material for: Comparative transcriptomic responses of tomato to Xanthomonas strains isolated from different hosts
Source: PLoS One. 2026 Nov 2;21(11):e0361370. doi: 10.1371/journal.pone.0361370 (PMC13726924; doi:10.1371/journal.pone.0361370)
Supplement: Supplementary file 1 [file pone.0361370.s003.docx]

SUPPLEMENTARY MATERIAL

| Sample | Total Bases | Total Reads | GC ratio (%) | Q_20_ (%) | Q_30_ (%) |
| --- | --- | --- | --- | --- | --- |
| Control_P1 | 6083941906 | 40291006 | 43.32 | 97.61 | 93.5 |
| Control_P2 | 9009087632 | 59662832 | 42.88 | 97.65 | 93.61 |
| Control_P3 | 6630818606 | 43912706 | 43.05 | 97.35 | 93.06 |
| INF_Xe_P1 | 6496815166 | 43025266 | 45.54 | 97.24 | 92.78 |
| INF_Xe_P2 | 8232888742 | 54522442 | 42.81 | 97.48 | 93.23 |
| INF_Xe_P3 | 8547393354 | 56605254 | 42.75 | 97.42 | 93.24 |
| INF_Xv_P1 | 8495035312 | 56258512 | 43.17 | 97.43 | 93.21 |
| INF_Xv_P2 | 8368552276 | 55420876 | 43.06 | 97.51 | 93.32 |
| INF_Xv_P3 | 8373579670 | 55454170 | 42.87 | 97.7 | 93.57 |

Table 1 raw throughput
