# Supplementary material for: Comparative transcriptomic responses of tomato to Xanthomonas strains isolated from different hosts
Source: PLoS One. 2026 Nov 2;21(11):e0361370. doi: 10.1371/journal.pone.0361370 (PMC13726924; doi:10.1371/journal.pone.0361370)
Supplement: Supplementary file 2 [file pone.0361370.s004.docx]

| Sample | Total Bases | Total Reads | GC ratio (%) | Q_20_ (%) | Q_30_ (%) |
| --- | --- | --- | --- | --- | --- |
| Control_P1 | 5745393097 | 39447326 | 43.17 | 98.41 | 94.63 |
| Control_P2 | 8616261883 | 58351392 | 42.8 | 98.43 | 94.74 |
| Control_P3 | 6199375246 | 42794620 | 42.82 | 98.33 | 94.43 |
| INF_Xe_P1 | 6095843744 | 41899620 | 45.37 | 98.2 | 94.15 |
| INF_Xe_P2 | 7849219050 | 53299460 | 42.7 | 98.31 | 94.4 |
| INF_Xe_P3 | 8134710790 | 55168632 | 42.68 | 98.35 | 94.53 |
| INF_Xv_P1 | 7960421110 | 54912514 | 42.99 | 98.34 | 94.49 |
| INF_Xv_P2 | 7872601092 | 54191194 | 42.84 | 98.36 | 94.53 |
| INF_Xv_P3 | 7915175427 | 54419326 | 42.65 | 98.4 | 94.59 |

Table 2 Clean throughput
