# Supplementary material for: Comparative transcriptomic responses of tomato to Xanthomonas strains isolated from different hosts
Source: PLoS One. 2026 Nov 2;21(11):e0361370. doi: 10.1371/journal.pone.0361370 (PMC13726924; doi:10.1371/journal.pone.0361370)
Supplement: Supplementary file 3 [file pone.0361370.s005.docx]

| Sample | Processed Reads | Mapped Reads | Mapping Ratio | Unmapped Reads | Unmapped Ratio |
| --- | --- | --- | --- | --- | --- |
| Control_P1 | 39447326 | 38764563 | 98.27% | 682763 | 1.73% |
| Control_P2 | 58351392 | 55836513 | 95.69% | 2514879 | 4.31% |
| Control_P3 | 42794620 | 41408100 | 96.76% | 1386520 | 3.24% |
| INF_Xe_P1 | 41899620 | 35286733 | 84.22% | 6612887 | 15.78% |
| INF_Xe_P2 | 53299460 | 52323722 | 98.17% | 975738 | 1.83% |
| INF_Xe_P3 | 55168632 | 54342147 | 98.5% | 826485 | 1.5% |
| INF_Xv_P1 | 54912514 | 53722454 | 97.83% | 1190060 | 2.17% |
| INF_Xv_P2 | 54191194 | 53032224 | 97.86% | 1158970 | 2.14% |
| INF_Xv_P3 | 54419326 | 53344680 | 98.03% | 1074646 | 1.97% |

Table 3 mapping
